# Selective Removal of Hemicellulose by Diluted Sulfuric Acid Assisted by Aluminum Sulfate

**DOI:** 10.3390/molecules29092027

**Published:** 2024-04-28

**Authors:** Huabin Jiang, Jiaqi Nie, Lei Zeng, Fei Zhu, Zhongwang Gao, Aiping Zhang, Jun Xie, Yong Chen

**Affiliations:** 1 Institute of Biomass Engineering, Key Laboratory of Energy Plants Resource and Utilization, Ministry of Agriculture and Rural Affairs, Guangdong Engineering Technology Research Center of Agricultural and Forestry Biomass, South China Agricultural University, Guangzhou 510642, China; jianghuabin12@163.com (H.J.); 18750127950@163.com (L.Z.); z20212149023@163.com (F.Z.); gaozw@scau.edu.cn (Z.G.); chenyong@ms.giec.ac.cn (Y.C.); 2 College of Materials and Energy, South China Agricultural University, Guangzhou 510642, China; aprilnjq@163.com

**Keywords:** hemicellulose, selective, cellulase, acid pretreatment

## Abstract

Hemicellulose can be selectively removed by acid pretreatment. In this study, selective removal of hemicellulose was achieved using dilute sulfuric acid assisted by aluminum sulfate pretreatment. The optimal pretreatment conditions were 160 °C, 1.5 wt% aluminum sulfate, 0.7 wt% dilute sulfuric acid, and 40 min. A component analysis showed that the removal rate of hemicellulose and lignin reached 98.05% and 9.01%, respectively, which indicated that hemicellulose was removed with high selectivity by dilute sulfuric acid assisted by aluminum sulfate pretreatment. Structural characterizations (SEM, FTIR, BET, TGA, and XRD) showed that pretreatment changed the roughness, crystallinity, pore size, and functional groups of corn straw, which was beneficial to improve the efficiency of enzymatic hydrolysis. This study provides a new approach for the high-selectivity separation of hemicellulose, thereby offering novel insights for its subsequent high-value utilization.

## 1. Introduction

Fossil fuels are a non-renewable source of energy, and excessive use has resulted in resource depletion, serious environmental pollution, and energy crises [1,2]. In order to alleviate the harm caused by environmental pollution and energy crises, the search for a green, renewable, and clean alternative has attracted increasing research interest [3]. Lignocellulose is an abundant, renewable, and readily degradable renewable energy source in nature, which can be converted into a variety of bioenergy and bio-based products, making it an ideal alternative to fossil energy sources [4,5].

Corn stover is a major crop in northern China, which is a huge resource to be developed [6]. However, it produces a large amount of stover waste every year. Due to the lack of effective utilization, landfills, in situ incineration, and other treatment methods are used, which not only pollute the environment, but also result in the waste of resources. Corn stover is mainly composed of cellulose, hemicellulose, and lignin [7]. Hemicellulose and lignin interact with cellulose through covalent/non-covalent bonds, constituting the “stubborn” structure of corn stover, which hinders degradation and significantly affects the efficiency of enzymatic saccharification [8]. To realize the efficient utilization of corn stover, it is first necessary to pretreat to break the “stubborn” structure of corn stover [9]. Among the three components, due to its lower degree of polymerization, hemicellulose is more easily degraded in the pretreatment process. Therefore, hemicellulose often is graded firstly, leaving as much lignin and cellulose left in the solids as possible [10,11].

Hemicellulose has characteristics such as gelling, emulsifying, and thermal chemical sensitivity. Its solubility and chemical activity are higher than cellulose, so it has shown certain potential applications in many fields and is used to produce many high value-added products [12]. In the field of chemical synthesis, hemicellulose is broken down and converted into chemical products such as xylose, ethanol, acetone, and butanol. Additionally, it serves as a common additive in the pulp and paper industry to enhance the tear resistance and other properties of paper, where a certain amount of hemicellulose is usually added to the pulp. Oligoxylose can improve the function of digestive systems in humans and animals, and hence, it is often used in food and animal feed. In the pharmaceutical industry, hemicellulose is used to prepare medications such as cholesterol inhibitors, sedatives, and tablet disintegrants. It also plays a crucial role in the prevention and treatment of degenerative joint diseases [13].

Pretreatment technologies included four categories: physical pretreatment, chemical pretreatment, physico-chemical pretreatment, and biological pretreatment. Among many pretreatment methods, acid pretreatment is one of the current large-scale application methods, which mainly dissolves and depolymerizes hemicellulose, destroys the rigid three-dimensional stubborn structure of corn stover, and can improve the accessibility of cellulase enzyme [14]. Liu et al. used oxalic acid pretreatment of poplar for the deconstruction and separation of hemicellulose. The hemicellulose separation was 79.08% in the optimized conditions [15]. Wang et al. used recyclable mandelic acid pretreatment for rapid and mild fractionation of hemicellulose, and 83.66% of xylose was separated under optimal conditions (temperature: 150 °C; concentration: 6.0%; time: 80 min) [16]. Previous studies have shown that acid pretreatment effectively disassembles lignocellulose, destroys the rigid structure of lignocellulose, and selectively removes hemicellulose. However, the hemicellulose removal rate was not satisfactory. Sulfuric acid is a strong acid and excessive use can corrode equipment and increase operating costs.

Aluminum sulfate is a cheap, low-toxicity, efficient, and stable metal salt catalyst for the efficient conversion of sugar and biomass-derived polymers [17]. The hydrolysis of aluminum sulfate produces Lewis acid and Bronsted acid, which can effectively assist in the bioconversion of corn stover fractions. Previous studies have shown that water and aluminum sulfate act synergistically to promote hemicellulose conversion [18]. However, the synergistic effect of aluminum sulfate and dilute acids and their role in pretreatment has not been reported.

In this study, a mixture of dilute sulfuric acid (DA) and aluminum sulfate (E520) was used as a corn stover pretreatment system. The influencing factors (aluminum sulfate concentration, pretreatment temperature, and time) of sulfuric acid and aluminum sulfate pretreatment were investigated. The microstructure, functional group changes, thermal stability, and crystal structure were investigated by SEM, FTIR, XRD, TGA, and BET tests. Due to the selective removal of hemicellulose and by increasing the porosity of the pretreated materials, it was expected that the combined pretreatment would improve the enzymatic hydrolysis effect by breaking through the rigid structure of corn stover. Finally, the mechanism of the combined pretreatment on corn stover was revealed.

## 2. Results and Discussion

### 2.1. Compositional Analysis of Solid Residue of DA/E520

**Figure 1:**
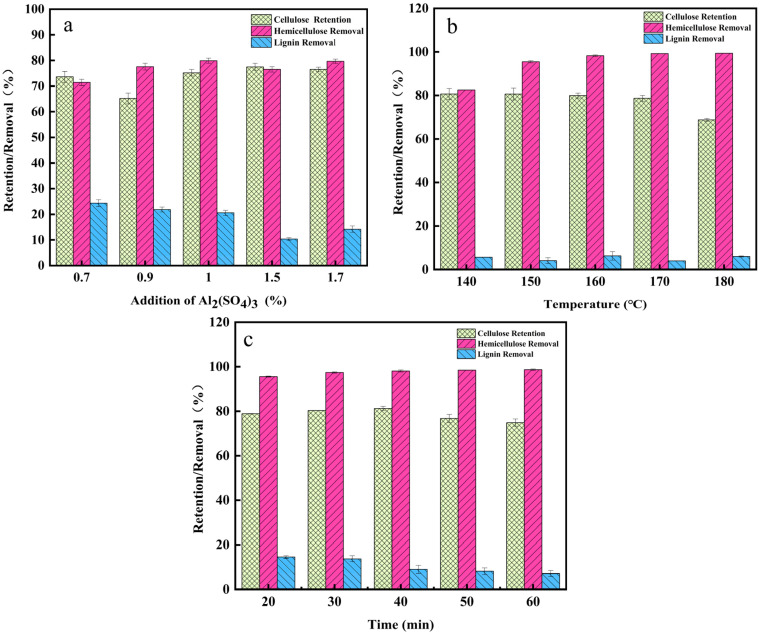
Removal of pretreatment fractions under dilute sulfuric acid conditions (140 °C, 40 min and different E520 addition levels) (**a**); removal of pretreatment fractions at different temperatures (**b**); removal of pretreatment fractions at different times (**c**); removal of components by different solvent pretreatment reactions.

Previous studies have shown that aluminum sulfate can improve the selective separation of hemicellulose [18]. In order to study the effect of aluminum sulfuric synergistic sulfuric acid pretreatment, different aluminum sulfuric concentrations (0.7 wt%, 0.9 wt%, 1.0 wt%, 1.5 wt%, and 1.7 wt%) were set. Previous studies have shown that the concentration of dilute sulfuric acid at 0.7 wt% has the best hemicellulose removal rate. The result is shown in Figure 1 and Table 1. From Table 1, it can be observed that the solid recovery of the residue decreased slowly when the aluminum sulfate content increased from 0.7% to 1.7%; this was because with the increase in E520 concentration, the synergistic effect of DA and E520 was enhanced, and more lignocellulose components were removed, resulting in less solid recovery [19]. The xylan content decreased slowly from 9.61% to 6.87%, which indicates that hemicellulose was removed in the pretreatment. The possible reason was that with the increase in E520 concentration, more hydrogen ions generated by hydrolysis of aluminum sulfate enhance the breaking of lignocellulosic glycosidic bonds [20]. Previous studies have shown that Al_2_(SO_4_)_3_ hydrolysis produced H^+^, [Al(OH)_2_(H_2_O)]^+^ and SO_4_^2−^. [Al(OH)_2_(H_2_O)]^+^ as a Lewis acid promoted the selective solubilization of hemicellulose, and H^+^ can selectively promote the conversion of hemicellulose to xylose [18]. Compared with other studies, we significantly improved the removal rate of hemicellulose.

Figure 1a shows the results of different aluminum sulfate concentrations assisted by DA on corn stover fractions. From Figure 1a, it can be observed that the cellulose yield increased slightly with the increase in aluminum sulfate content; hemicellulose removal was the first to increase from 71.47% to 80.51%, and then slowly decreased to 76.54%; and the removal rate of lignin decreased from 24.36% to 10.35% with the gradual increase in aluminum sulfate addition in the pretreatment, which indicated that the addition of aluminum sulfate inhibited the removal of lignin. These results showed that the combination pretreatment with aluminum sulfate achieved efficient and selective removal of hemicellulose while inhibiting the dissolution and conversion of lignin and cellulose. At 1.5% aluminum sulfate addition, although the removal rate of lignin was 10.35%, the retention rate of cellulose was 77.49%, and the removal rate of hemicellulose was 76.54%, so in order to improve the efficiency of the subsequent enzymatic hydrolysis, the addition of aluminum sulfate in the subsequent experiments was selected as 1.5%.

Figure 1b shows the effect of different temperature conditions on corn stover fractions. From Table 1, it can be observed that when the temperature increased from 140 °C to 180 °C, the solid recovery of the residue gradually decreased to 56.25%. The separation effect of hemicellulose was increased due to the increased efficiency of mass and heat transfer under high temperature conditions [21]. The content of xylan gradually decreased, and the relative content of lignin showed an increasing trend. As shown sin Figure 1b, the glucose removal rate increased from 19.51% to 31.22% with the increase in pretreatment intensity, indicating that the degradation of cellulose was strengthened by aluminum sulfate-catalyzed dilute sulfuric acid pretreatment, and the retention rate of cellulose decreased. For hemicellulose, with the increase in temperature, the hemicellulose removal rate increased rapidly from 82.54% to 99.36%, indicating that temperature changes on hemicellulose had a greater impact on the hemicellulose [22]. The result showed that the combination of pretreatments on hemicellulose removal has a better selectivity. Compared with the single dilute sulfuric acid pretreatment, there was a significant decrease in lignin removal with the addition of aluminum sulfate. At this pretreatment temperature of 160 °C, the cellulose yield rate was 79.91%, and hemicellulose was almost removed, with a removal rate of 98.24%.

As shown in Table 1, when pretreatment time was increased from 20 min to 50 min, the glucose content was maintained in the range of 46.09–48.38% and the cellulose yield was in the range of 76.71–81.25%. From Figure 1c, it can be observed that the removal of hemicellulose remained higher, around 97%, and the increase in time had a minimal enhancement on the removal. When the pretreatment time was 40 min, the cellulose yield was optimal (81.25%) and the hemicellulose removal reached 98.05%, at which time the lignin removal was only 9.01%.

A comparative study with other selective removal and pretreatment techniques of hemicellulose was carried out. The results are presented in Supplementary Materials Table S1. Compared with other pretreatment techniques, the pretreatment combination of aluminum sulfate with dilute sulfuric acid used in this study obtained the highest hemicellulose removal rate.

### 2.2. Compositional Analyses Liquid Fractions

The glucose and xylose produced during dilute sulfuric acid pretreatment are degraded to 5-hydroxymethylfurfural and furfural, while acetyl groups in hemicellulose are degraded and produce acetic acid, and furans in lignin, as well as phenolic compounds [23]. As shown in Table 2, the reaction at all temperatures produced only very little furfural and almost no 5-hydroxymethylfurfural, indicating that the temperature of this experiment was mild and all pretreatment conditions were not sufficient to degrade glucose to produce 5-hydroxymethylfurfural. Compared to 5-hydroxymethylfurfural and furfural, formic acid and acetic acid were the main inhibitors produced during the dilute sulfuric acid pretreatment, suggesting that xylose is more susceptible to degradation than glucose [24].

As the pretreatment conditions were further intensified, the concentrations of various types of inhibitors in the pretreatment solution gradually increased, indicating the generation of more inhibition products [25]. In the time-adjusted one-way experiments, the inhibitor content under each treatment condition showed a slow increase with the extension of time. In the concentration experiments, when the pretreatment conditions were 140 °C and 40 min, and the solvent concentration was 0.5%, the concentration of 5-hydroxymethylfurfural was only 0.01 g/L, furfural concentration was 0.15 g/L, formic acid concentration was 0.25 g/L, and acetic acid concentration was 0.24 g/L. When the pretreatment concentration was increased to 1.0%, the concentration of 5-hydroxymethylfurfural was 0.02 g/L, and the concentration of 5-hydroxymethylfurfural was 0.28 g/L. The concentration of the inhibitor in the time-adjusted one-way experiments was 0.28 g/L. The inhibitor content in the time-adjusted one-way experiments increased slowly with time. When the pretreatment concentration was increased to 1.0%, the concentration of 5-hydroxymethylfurfural was 0.02 g/L, the concentration of furfural was 0.28 g/L, the concentration of formic acid was 0.28 g/L, and the concentration of acetic acid was 0.33 g/L. The concentration of sulfuric acid had a greater effect on the production of furfural, formic acid, and acetic acid, and the concentrations of the three major inhibitors increased with the increase in sulfuric acid concentration, which was consistent with the corresponding xylan content in Table 1, which decreased gradually with the intensification of the reaction conditions.

As shown in Table 2, xylose was the main sugar present in the pretreatment solution, indicating that hemicellulose was degraded substantially in the DA/E520 pretreatment under different conditions, with most of the cellulose being retained in the pretreatment residue. For xylose, the xylose content in the treatment solution increased with the addition of aluminum sulfate, with the total xylose content reaching a maximum of 20.12 g/100 g at 1.5% aluminum sulfate addition, with 88.32% of the total xylose, indicating that dilute acid pretreatment was beneficial to the release of xylose [26]. When the temperature was gradually increased from 140 °C to 180 °C, the total xylose increased from 20.24 g/100 g to 21.27 g/100 g. The xylose content in the pretreatment solution showed a slow growth trend, indicating that the degradation of hemicellulose was slowed down. The increase in pretreatment time had minimal effect on the removal of hemicellulose and lignin, with the xylose content reaching 21.15 g/100 g at 40 min.

Due to the acidity, some of the glucose and xylose produced during dilute sulfuric acid pretreatment of corn stover was degraded to 5-hydroxymethyl furfural and furfural, while acetyl groups in hemicellulose were degraded and acetic acid was produced, and furans in lignin and phenolic compounds were degraded to release formic acid. Therefore, it was necessary to analyze the content of the inhibitors produced by the pretreatment solution.

The low concentrations of both 5-HMF and furfural in the dilute sulfuric acid and sulfuric acid/glycol pretreatment filtrates indicate that cellulose is less degraded during the pretreatment process. The main inhibitors were acetic acid from hemicellulose degradation and formic acid from lignin degradation. In the Supplementary Materials, for dilute sulfuric acid with the addition of different levels of aluminum sulfate, the single-factor inhibitor content changes. With increasing levels of addition, 5-HMF just varied between 0.02 g/L and 0.03 g/L. The content of furfural, formic acid, and acetic acid increased correspondingly with the increase in the added amount in the filtrate. The 5-HMF, furfural, acetic acid, and formic acid content in the pretreatment filtrate at each temperature showed a rapid increase with increasing temperature. 5-HMF gradually increased from a concentration of 0.05 g/L to 0.99 g/L; furfural increased from 0.54 g/L to 1.94 g/L; formic acid increased rapidly from 0.24 g/L to 12.91 g/L; and acetic acid increased from 0.26 g/L to 1.71 g/L. These data further illustrate the large effect of temperature changes on the reaction and on the production of inhibitors a significant effect. At a reaction temperature of 160 °C and different reaction times, both of the four inhibitors increased slowly with prolonged reaction time.

### 2.3. Enzymatic Hydrolysis of Different Pretreatment Substrate

**Figure 2:**
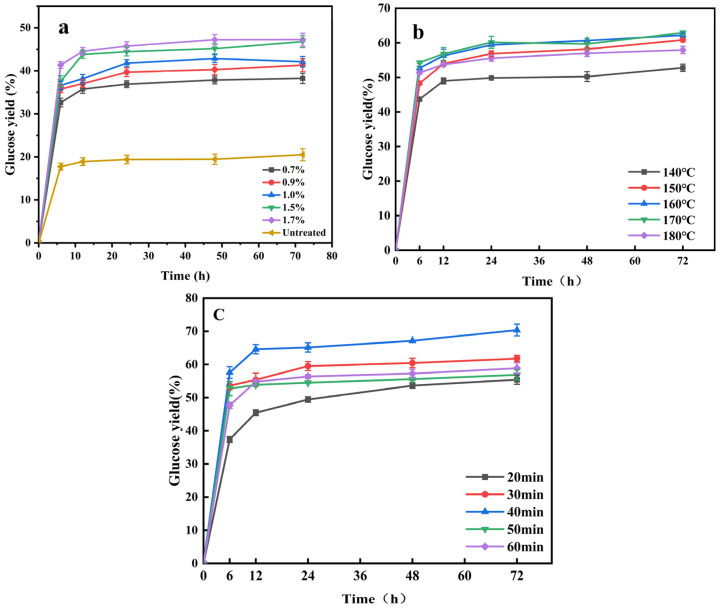
Glucose yield after DA/E520 pretreatment at different E520 addition (**a**); glucose yield after DA/E520 pretreatment with different temperature (**b**); glucose yield DA/E520 pretreatment with different times) (**c**).

In order to study the enzymatic hydrolysis potential of the pretreatment products, the effects of different pretreatment factors (concentration, temperature, and time) in DA/E520 pretreatment on the enzymatic effect were investigated as shown in Figure 2. As shown in Figure 2a, the unpretreated corn stover had a glucose yield of 20.5% after 72 h of enzymatic digestion. This is because the stubborn structure of the corn straw resisted the degradation of cellulase. However, the results of concentration optimization showed that with the increase in aluminum sulfate, the glucose yield gradually increased. The pretreatment reaction removes little cellulose and most of hemicellulose, destroys the lignocellulosic structure and improves cellulase action with cellulose [27]. When concentrations of 0.7%, 0.9%, 1.0%, 1.5%, and 1.7% aluminum sulfate were added, the glucose yield reached 38.26%, 41.32%, 42.13%, 46.79%, and 47.26%. The maximum glucose yield was increased two-fold compared with the raw corn stover. The difference between the enzymatic yield at 1.5% and 1.7% was minimal.

The effect of different pretreatment temperatures on glucose yield was further studied. A clear increase in glucose yield with increasing temperature can be seen in Figure 2b. The yield of glucose increased gradually with the time of enzymatic hydrolysis until it reached stability. When the pretreatment temperature increased from 140 °C to 170 °C, the glucose yield gradually increased. The highest glucose yield was 62.89%. The reason for this is that with the increase in the degree of pretreatment, the components of lignocellulose were separated and dissolved, exposing the cellulose framework and more active sites for cellulose enzyme binding, thereby enhancing the production of glucose [28]. However, when the pretreatment temperature was 180 °C, the glucose yield was only 52.92%. The excessive intensity of pretreatment caused the structure of corn straw to be seriously damaged (for example, the specific surface area is reduced, the aperture collapses, and so on) and the efficiency of enzymatic hydrolysis was reduced [15].

The pretreatment time can affect the severity factor and affect the glucose yield. Therefore, the effect of pretreatment time (20–60 min) on the enzymatic hydrolysis of carbohydrate was studied. Figure 2c shows the variation in the effect of different pretreatment times on glucose yield. With the increase in pretreatment time, the glucose yield of different pretreatment substrates increased significantly. Different pretreatment times had different effects on glucose yield. When the pretreatment time increased from 20 min to 40 min, the glucose yield gradually increased to the maximum, reaching 70.37%. However, as the pretreatment time increased from 40 min to 60 min, glucose production began to decline. This may be because as the pretreatment time increased, more acidic substances reached the interior of the corn stover, enabling efficient dismantling. So cellulase breaks down cellulose to produced more glucose. However, with the increase in pretreatment time, the structure of corn straw was seriously damaged, and more cellulose was removed, resulting in a decrease in glucose yield.

Therefore, the optimum reaction conditions for the dilute aluminum sulfate pretreatment were as follows: 0.7% sulfuric acid combined with 1.5% aluminum sulfate, reaction temperature 160 °C and reaction time 40 min, taking into account the inhibitor content of the component changes and the glucose yield of the enzymatic digestion under each single factor.

### 2.4. Characterization

**Figure 3:**
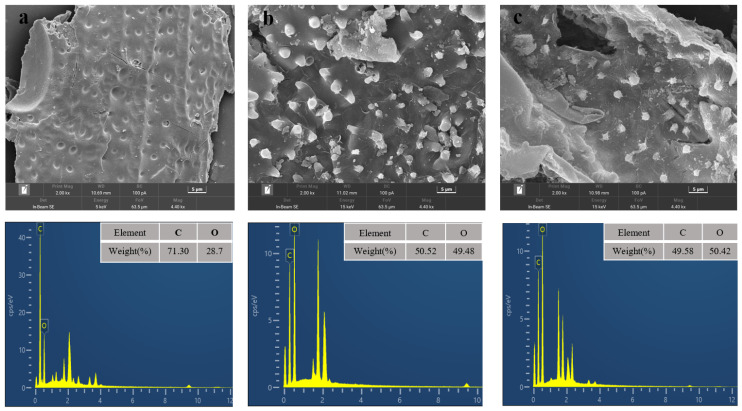
SEM-DES of raw material (**a**); after pretreatment (0.7 wt% H_2_SO_4_, 1.5 wt% Al_2_(SO_4_)_3_, 140 °C, 40 min) (**b**); after pretreatment (0.7 wt% H_2_SO_4_, 1.5 wt% Al_2_(SO_4_)_3_, 160 °C, 40 min) (**c**).

In order to understand the effect of pretreatment process on corn stover, some characterization methods such as SEM, FTIR, XRD, etc., have been applied to enhance the understanding of pretreatment. SEM-EDS maps of corn stover were analyzed and the results are shown in Figure 3. From the SEM, it can be seen that the surface of the corn stover was smooth, and the fibers were longitudinally and transversely intertwined to form a recalcitrant structure to prevent the degradation of cellulase [29]. After pretreatment, the surface of corn straw appeared to fold and hollow structure and many fine particles existed. This may be that a large amount of hemicellulose was removed during pretreatment and the dense structure was destroyed. With the end of the reaction, part of the lignin condensed on the surface, forming pseudo-lignin. The loose structure after pretreatment helps to improve the accessibility of cellulase, make cellulase easy to hydrolyze, and improve the hydrolysis efficiency [30].

FTIR can show the changes in lignocellulosic functional groups before and after pretreatment. The FTIR results of different pretreatment samples are shown in Figure S1 (see Supplementary Materials). The board band at 3405 cm^−1^ was attributed to the O-H stretching effect of the cellulose macromolecules [31]. The peak intensity had no significant change before and after pretreatment. This indicated that cellulose was retained after pretreatment. The band at 897 cm^−1^ is characteristic of the glycosidic β-(1→4) bond in cellulose [32]. The peaks at 1417 cm^−1^, 1384 cm^−1^ and 1321 cm^−1^ belong to crystalline cellulose. The characteristic peaks of C=O and C-O bonds appeared in 1736 cm^−1^ and 1244 cm^−1^, respectively [21]. After pretreatment, the peak became smaller and almost disappear, which was due to the selective removal of hemicellulose. The characteristic peaks of the lignin aromatic skeleton appeared at 1512 cm^−1^ and 1651 cm^−1^ [31]. Compared with RM, the peak strength after pretreatment increased, which was due to the separation of hemicellulose [24].

The thermal stability of pretreated samples varied with separation of three components [16]. The results of TG are shown in Figure S2 (see Supplementary Materials). The main weight loss was concentrated in the thermal degradation of cellulose. After pretreatment with different concentrations of aluminum sulfate, the cellulose content increased from 53.84% (RM) to 62.66% (1 wt%), 63.14% (1.5 wt%), and 59.99% (1.7 wt%), respectively. The results showed that they had similar relative cellulose content. However, 1.5 wt% aluminum sulfate addition had the greatest weight reduction and showed better cellulose yield ability. This result was also consistent with the enzymatic hydrolysis effect mentioned above.

XRD analysis was used to analyze the change in substrate crystallinity before and after pretreatment. Sample crystallinity affects the efficiency of enzymatic hydrolysis [33]. As shown in Figure S3 (see Supplementary Materials), the pretreated corn stover had higher crystallinity. The crystallinity of corn stover was only 29.11%. However, due to the removal of hemicellulose, the crystallinity of the samples increased after pretreatment [34].

The changes in specific surface area and pore size of the samples after pretreatments are shown in Figure S4 (see Supplementary Materials). After pretreatment, the pore sizes were reduced. The pore size was reduced from 26.6296 nm of raw material to 12.7201 nm after 1.5 wt% aluminum sulfate pretreatment. However, the pore volume increased significantly. In particular, the addition of 1.5 wt% aluminum sulfate increased the pore volume five-fold, from 5.082 dm^3^/g to 25.019 dm^3^. At the same time, the specific surface area changed from 1.1979 m^2^/g of raw material to a maximum of 5.9851 m^2^/g (1.5 wt%). The BET results showed that the addition of aluminum sulfate pretreatment could significantly increase the pore volume and specific surface area, which was conducive to improving the accessibility of cellulase and the enzymatic efficiency.

### 2.5. Mass Balance

**Figure 4:**
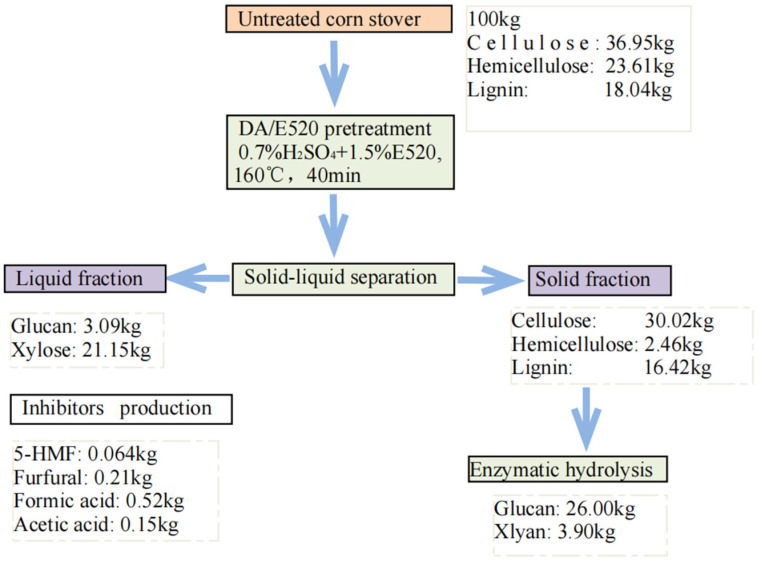
Mass balance analysis of DA/E520 and enzymatic hydrolysis procedures.

The mass balance was performed (optimum DA/E520 system (1.5% E520 addition, 160 °C and 40 min of treatment) for 100 kg of corn stover biomass and included values for cellulose, xylan, and lignin. The data in this analysis were obtained from the experimental results. The results of mass balance was shown in Figure 4. The raw materials mainly contain cellulose (36.95%), hemicellulose (23.61%), lignin (18.04%), and other components (21.40%). The results showed that 30.02 kg of cellulose (based on the initial untreated biomass) was retained in the solid fraction and approximately 3.09 kg of cellulose was hydrolyzed to glucose; and 16.42 kg of lignin and 2.46 kg of hemicellulose were still retained in the straw. The enzymatic digestibility of the cellulose solid fraction yielded a glucose yield of 26.0 kg, which means that 70.37% of the initial cellulose was converted to glucose. For lignin, the solid fraction of the biomass retained most of the lignin 16.42 kg. In addition, 21.15 kg of all quantities of xylose were extracted and dissolved in the liquid phase, with 2.46 kg retained in the solid fraction. The hydrolysis of hemicellulose in the liquid yielded 89.58% of total xylose. The remaining small fractions were degraded to inhibitors in the reaction, mainly furfural, formic acid, and acetic acid.

In this study, aluminum sulfate (1.7 wt%) and dilute sulfuric acid (0.7 wt%) were used to pretreat corn stover. The selective separation of hemicellulose using a dilute acid solution reduced the corrosion of the equipment and the overall process energy consumption. The addition of aluminum sulfate further enhanced the removal rate of hemicellulose, and realized the value of hemicellulose into high-value-added products. In addition to the efficient removal of hemicellulose, the solution can be recycled to reduce costs and reduce environmental pollution.

## 3. Materials and Methods

### 3.1. Materials

In this study, corn stover was used without removal of leaves and other parts. Corn stover was collected from Shandong Province, China, which was screened to 40–60 mesh and extracted with toluene/ethanol (2:1, *v*/*v*) in Soxhlet extractor for 6 h, and the extractive-free samples were oven-dried at 50 °C for 24 h. The chemical compositions of corn stover, which was composed of 36.95% cellulose, 23.61% hemicellulose, 18.04% lignin, 8.80% ash and 12.60% other components, were determined according to the National Renewable Energy Laboratory (NREL) method.

Reagents including all sugars (glucose, xylose), aluminum sulfate (E520) and dilute sulfuric acid (DA), formic acid (FA), acetic acid (AA), furfural (FF), and 5-hydroxymethylfurfural (5-HMF) were purchased from Aladdin Chemical Reagent Co., Ltd. (Shanghai, China). Hydrolysis enzymes of Cellic CTec2 with a filter paper activity (FPU) of 138 FPU/mL in this work was purchased from Novozymes (Glendale, CA, USA) [35].

### 3.2. Pretreatment of Corn Stover

The well-dried corn powders were loaded into the reactor and blended well with 140 mL of solvent solution. The DA/E520 pretreatment at solid-to-liquid ratio of 1:20 was carried out at various E520 concentrations (0.9, 1.0, 1.3, 1.5, and 1.7%), temperatures (140, 150, 160, 170, and 180 °C) and times (20, 30, 40, 50, and 60 min). In addition, pretreatment of DA was conducted at the identified optimized conditions as a comparison. After cooling down the reactor, cellulose-rich solid residues were separated. Solid residues were rinsed several times with deionized water till neutralization of pH, then kept at 4 °C for further analysis and enzymatic digestibility [33].

### 3.3. Analysis of Solid Residue and Liquid Fraction

Pretreatment solid recovery yield (PSY), cellulose yield, lignin removal, and hemicellulose removal were estimated as shown in Equations (1)–(4);

(1) $$\mathrm{PSY}\;(\%)=\frac{Pretreated\;residue\;(g)}{Biomass\;weight\;(g)}\times 100$$

(2) $$\mathrm{Cellulose}\;\mathrm{yield}\;(\%)=\frac{Cellulose\;amount\;in\;pretreated\;residue\;(g)}{Cellulose\;amount\;before\;preatment\;(g)}\times 100$$

(3) $$\mathrm{Lignin}\;\mathrm{removal}\;(\%)=(1-\frac{Lignin\;amount\;in\;pretrented\;residue\;(g)}{Lignin\;amount\;before\;pretreatment\;(g)})\times 100$$

(4) $$\mathrm{Hemicellulose}\;\mathrm{removal}\;(\%)=(1-\frac{hemicellulose\;amount\;in\;pretreated\;residue\;(g)}{hemicellulose\;amount\;before\;pretreatment\;(g)})\times 100$$

The determination of glucose and xylose content in the pretreatment solution and enzymatic digestion solution requires the degradation of oligosaccharides to monosaccharides firstly, and then calculated by the increase in monosaccharides [36]. The glucose and xylose contents were measured by HPLC-20AD (Shimadzu, Kyoto, Japan) [37]. HPLC uses Shodex KS-801 column with a flow rate of 0.4 mL/min and a column temperature of 60 °C. HPLC uses pure water as the mobile phase. The chromatographic column (Shodex KS-801) is provided by Guangzhou Green Baicao Scientific Instrument Co., Ltd. (Guangzhou, China). Following that, a UV spectrophotometer was used to determine acid-soluble lignin (ASL) at 320 nm, while acid insoluble lignin (AIL) was calculated from the mass of solids after acid hydrolysis minus ash [38]. The inhibitors from the further degradation of monosaccharides in the pretreatment solution were determined by high-performance liquid chromatography (HPLC) using a C-610H column with an ultraviolet detector (SPD-20A) at a detection wavelength of 210 nm. The mobile phase was 0.1% phosphoric acid after degassing. The flow rate was 0.7 mL/min, and the column temperature was 35 °C. The sample size was 10 μL, and the detection operation time was 90 min [39].

### 3.4. Solid Residue Characterization

The chemical bonding changes in the corn stover feedstock and pretreated samples were measured by a vertex 70 Fourier Transform Infrared spectrometer (FTIR). The specific surface area was determined using the BET method. SEM was used to analyze the microscopic physical morphological changes. The thermal stability was assessed by TGA.

The crystallinity index (CrI) was determined XRD. The equation is as follows:

(5) $$\mathrm{CrI}=\frac{I_{002}-I_{\mathrm{am}}}{I_{002}}\;\times \;100\%$$

### 3.5. Enzymatic Hydrolysis

An amount of 2.00 g of treated residue was weighed into a 150 mL conical flask and 100 mL (pH = 4.8) citric acid-trisodium citrate buffer solution was added to give a solids load of 2% (*w*/*v*), followed by 15 FPU/g dry matter of cellulase. The conical flask was fixed smoothly in a thermostatic shaker, set at 150 r/min and 50 °C. The enzymatic digestion was carried out for 72 h. The glucose concentration was determined by HPLC [40]. Glucose yield was estimated as shown in Equation (6); 0.9 is the coefficient between glucose and cellulose.

(6) $$\mathrm{Glucose}\;\mathrm{yield}\;(\%)=\frac{Glucose\;produced\;(g)\times 0.9}{Cellulose\;content\;in\;Raw\;material\;(g)}\times 100\%$$

## 4. Conclusions

In this study, the performance of aluminum sulfate-assisted dilute sulfuric acid pretreatment of corn stover was evaluated. In addition, the nature of cellulose in the residue, the variation in inhibitor content in the filtrate, and the subsequent enzymatic performance were also investigated. The pretreatment results showed that a combined pretreatment could remove hemicellulose with high selectivity. The characterization of the straw stover before and after pretreatment showed that the DA/E520 pretreatment was able to increase the specific surface area and improve the thermal stability of the straw stover. Under optimal pretreatment conditions, 98.05% hemicellulose removal, 81.25% cellulose yield, and 70.37% subsequent enzymatic yield of cellulose were achieved. This glucose yield was 11.21% higher compared to the DA pretreatments, respectively. In addition, the hydrolysis of hemicellulose in the liquid yielded 89.58% of total xylose. To conclude, this work presents an effective solution for the separation of hemicellulose that will contribute to the efficient conversion and utilization of biomass.

## Figures and Tables

**Table 1:** Pretreatment yield (PY) and chemical compositions of solid residues after pretreatments. Results as mean ± SD (n = 3).

Addition of E520	T °C	t min	PY (%)	Cellulose (%)	Xylan (%)	Lignin (%)
Untreated			-	36.95 ± 0.15	23.61 ± 0.06	18.04 ± 0.39
DA	140	40	55.97	59.62 ± 1.61	3.67 ± 0.87	27.02 ± 0.22
0.7%	140	40	70.10	24.64 ± 0.61	8.57 ± 0.21	19.14 ± 0.21
0.9%			70.58	38.81 ± 0.84	9.61 ± 0.16	19.47 ± 0.17
1.0%			67.62	34.12 ± 0.53	7.51 ± 0.10	19.97 ± 0.31
1.5%			68.90	40.23 ± 0.95	6.87 ± 0.05	20.75 ± 0.56
1.7%			66.92	41.55 ± 1.23	8.04 ± 0.01	23.47 ± 0.18
1.5%	150		61.66	48.30 ± 1.05	1.73 ± 0.17	28.06 ± 0.05
	160		61.07	48.85 ± 0.15	0.64 ± 0.15	27.42 ± 0.25
	170		59.61	48.77 ± 0.78	0.31 ± 0.02	29.04 ± 0.02
	180		56.25	45.18 ± 0.58	0.28 ± 0.12	30.14 ± 0.16
1.5%	160	20	61.32	47.52 ± 0.07	1.69 ± 0.08	25.14 ± 0.16
		30	61.46	48.31 ± 0.81	1.01 ± 0.10	25.33 ± 0.80
		40	61.07	48.85 ± 1.22	0.64 ± 0.17	27.42 ± 0.19
		50	61.47	46.09 ± 0.53	0.59 ± 0.02	26.96 ± 0.77
		60	62.89	43.96 ± 1.38	0.50 ± 0.13	26.64 ± 0.29

**Table 2:** Content of sugar and inhibitor in hydrolysate of corn stalk pretreated with dilute acid aluminum sulfate.

Pretreatment Condition			Sugar Content (g/100 g)				Inhibitor Content (g/L)			
DA+E520	T (°C)	t (min)	Total Glucose	Xylose	Xylan	Total Xylose	HMF	Furfural	Formic Acid	Acetic Acid
0.7% + 0.7% E520	140	40	3.51	13.60	4.93	18.53	0.02	0.11	0.19	0.09
0.7% + 0.9% E520			3.67	14.29	5.41	19.70	0.02	0.11	0.19	0.11
0.7% + 1.0% E520			3.54	16.45	2.57	19.02	0.03	0.18	0.16	0.14
0.7% + 1.5% E520			3.39	17.77	2.35	20.12	0.03	0.24	0.22	0.17
0.7% + 1.7% E520			3.47	16.94	3.16	20.10	0.03	0.27	0.26	0.17
0.7% + 1.5% E520	140	40	3.39	17.77	2.35	20.12	0.03	0.24	0.22	0.17
	150		3.16	17.27	2.34	19.61	0.15	1.26	1.07	0.47
	160		3.34	19.44	1.68	21.12	0.38	1.87	3.50	1.11
	170		3.41	20.02	1.21	21.23	0.71	1.94	8.45	1.74
	180		3.45	19.99	1.28	21.27	0.99	1.76	12.91	1.71
0.7% + 1.5% E520	160	20	3.14	15.98	4.37	20.35	0.24	1.45	1.66	0.75
		30	3.11	14.60	5.51	20.11	0.33	1.69	2.46	0.95
		50	3.23	17.05	4.14	21.19	0.53	1.96	4.54	1.22

## Data Availability

Data are contained within the article and Supplementary Materials.

## Supplementary Materials

The following supporting information can be downloaded at: https://www.mdpi.com/article/10.3390/molecules29092027/s1, Table S1: Hemicellulose was removed by different pretreatment techniques; Table S2: Effect of dilute acid sulfuric acid pretreatment on chemical composition change; Figure S1: FTIR of RM and different pretreatment samples; Figure S2: TG of RM and different pretreatment samples; Figure S3: XRD of RM and different pretreatment samples; Figure S4: BET of RM and different pretreatment samples; Table S3: The result of BET data. References [41,42,43,44] are cited in Supplementary Materials.
