# Supplementary material for: Selective Removal of Hemicellulose by Diluted Sulfuric Acid Assisted by Aluminum Sulfate
Source: Molecules. 2024 Apr 28;29(9):2027. doi: 10.3390/molecules29092027 (PMC11085920; doi:10.3390/molecules29092027)
Supplement: Supplementary file 1 [file molecules-29-02027-s001.zip › molecules-2973763-supplementary.pdf]

Selective removal of hemicellulose by diluted sulfuric acid as-sisted by aluminum sulfate

Jiang Huabin[1], Zeng Lei[1], Nie Jiaqi[2], Zhu Fei[1], Gao Zhongwang[1], Zhang Aiping[1]*, Xie Jun[1]*, Chen Yong[1]

1 Institute of Biomass Engineering, Key Laboratory of Energy Plants Resource and Utilization, Ministry of Agriculture and Rural Affairs, Guangdong Engineering Technology Research Center of Agricultural and Forestry Biomass, South China Agricultural University, Guangzhou 510642, China

2 College of Materials and Energy, South China Agricultural University, Guangzhou 510642, China

* Author to whom correspondence should be addressed

Table S1 Hemicellulose was removed by different pretreatment techniques

Pretreatment method	Raw materials	Condition	Hemicellulose removal	Reference
Mannitol, oxalic acid	Poplar	OA 7.0%, MA 7.0% 150 °C, 60 min	79.08%	[15]
Mandelic acid	Eucalyptus	6.0 wt%, 150 °C, 80 min	83.66%	[16]
Vanillic acid	Eucalyptus	8.0%, 170 °C, 80 min	88.59%	[10]
Tetramethylammonium hydroxide (TMAH)	Poplar	25wt%	66.4%	[41]
Benzoic acid (BZA)	Poplar	2%, 160 °C, 0.75 h	93.1%	[42]
Saturated steam	Wheat straw	180 °C, 10 bar, 35 min	63%	[12]
Microwave, choline chloride/p-TsOH	Reed	ChCl/p-TsOH(1:2), 100 °C, 30 min	89.41%	[43]
Press, Hydrothermal	Kraft pulping.	155 °C, 3MPa	70.63%	[44]
Aluminum sulfate, H_2SO_4	Straw stover	160 °C, 1.5wt%E520, 0.7 wt%DA, 40 min	98.05%	This study

Table S2 Effect of dilute acid sulfuric acid pretreatment on chemical composition change

Pretreatment conditions			Solid recovery（%）	Component content（%）		
Temperature (℃)	Time (min)	Concentration %		Glucan	Xylan	Lignin
Corn stover			—	36.95	23.61	18.04
100	30	0.5	84.68	42.95	21.86	20.14
110			73.76	46.79	15.73	21.36
120			66.71	49.37	11.81	22.24
130			62.96	55.21	7.94	23.91
140			57.60	58.39	4.63	24.77
140	20	0.5	61.86	55.46	6.77	23.85
	30		59.14	56.90	6.44	23.17
	40		59.53	57.31	5.36	24.34
	50		58.34	57.79	4.56	25.08
140	40	0.5	56.85	60.39	4.46	26.85
		0.7	55.97	59.62	3.67	27.02
		0.9	54.40	56.54	2.37	27.42
		1.0	53.83	48.87	1.31	28.40
		1.2	52.23	41.19	0.77	28.47

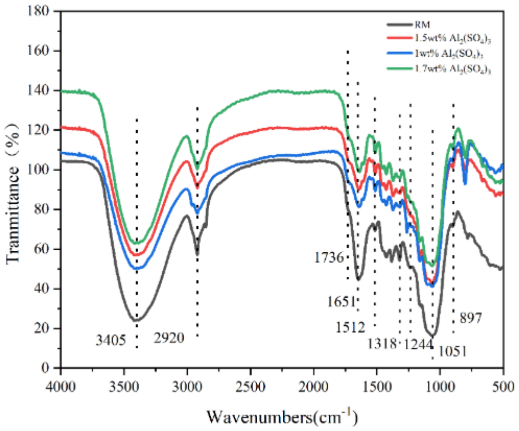

Fig. S1 FTIR of RM and different pretreatment samples

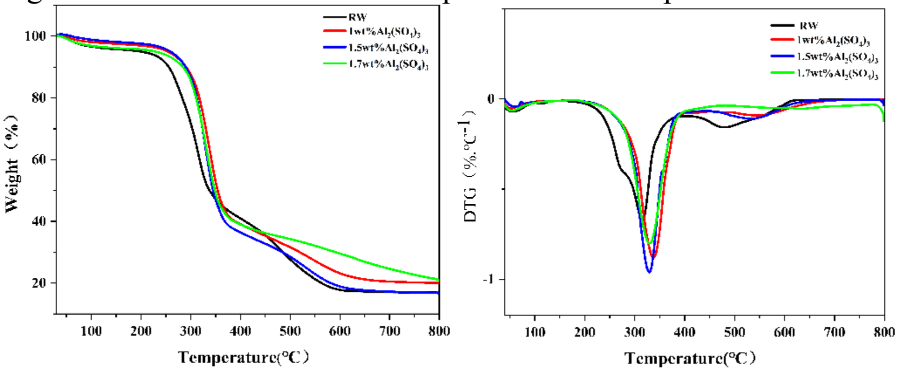

Fig.S2 TG of RM and different pretreatment samples

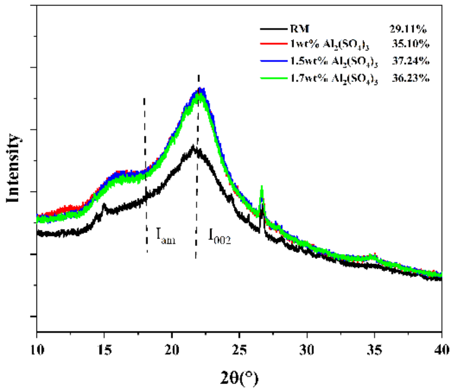

Fig.S3 XRD of RM and different pretreatment samples

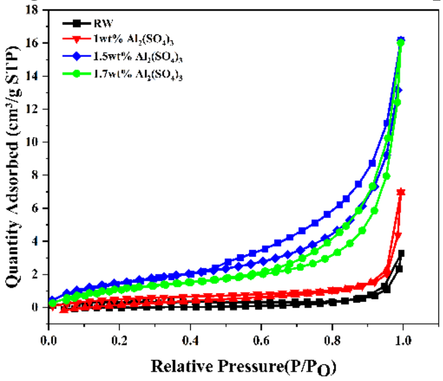

Fig.S4 BET of RM and different pretreatment samples

Table S3 The result of BET data

	BET Surface Area (m^2/g)	Pore Volume (dm^3/g)	Pore Size(nm)
RM	1.1979	5.082	26.6296
1wt%	2.1820	10.768	24.3572
1.5wt%	5.9851	25.019	12.7201
1.7wt%	4.6489	24.776	18.0721

Six chromatograms of glucose and xylose standard samples, 1 chromatogram of raw material components of corn stalk and 2 chromatographs of pretreated samples were presented. The peak retention times of glucose and xylose were 20.1 and 21.5, respectively.

0.3 g/L glucose and xylose(standards mixture)

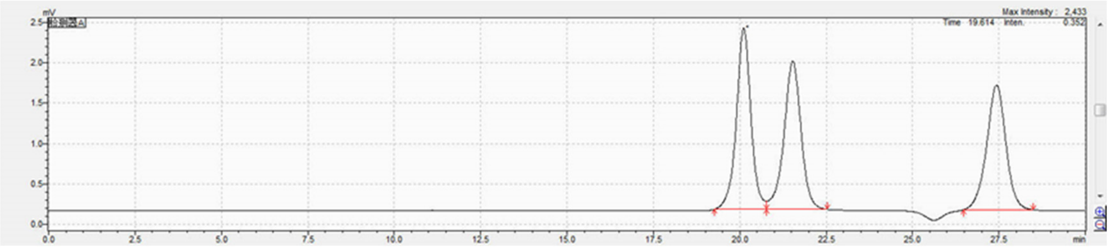

The raw material before pretreatment (Corn stalk)

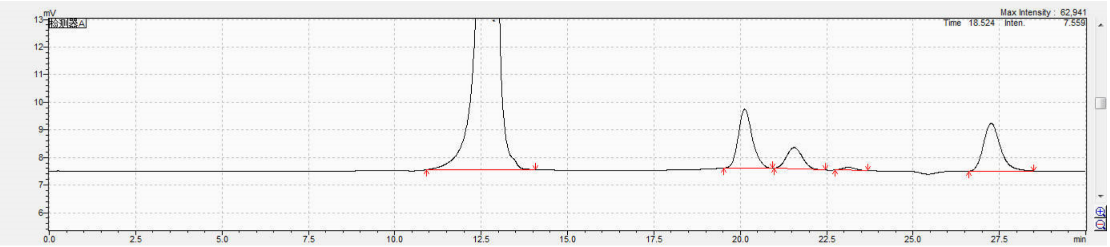

The sample 1 after pretreatment

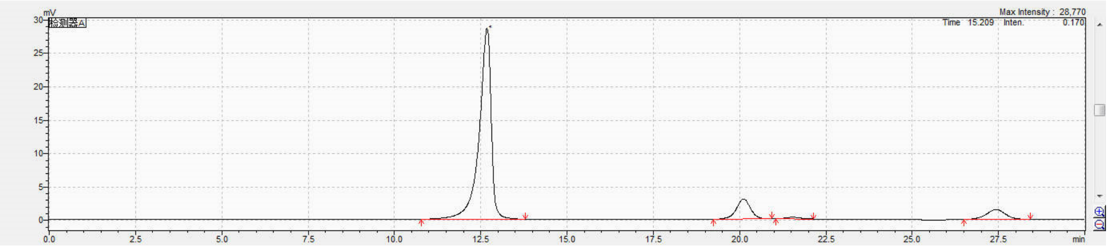

Standard curve chromatograms of inhibitors and sample chromatograms were presented. The peak retention times of formic acid, acetic acid, HMF and furfural were 19.85, 21.69, 53.14 and 80.01, respectively.

0.3 g/L formic acid, acetic acid, HMF and furfural (standards mixture)

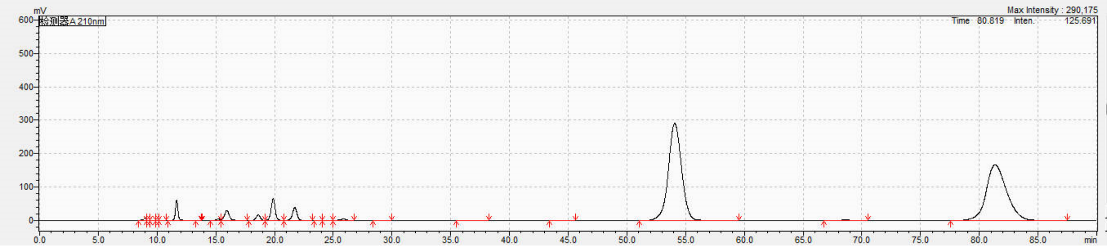

Sample1 after pretreatment

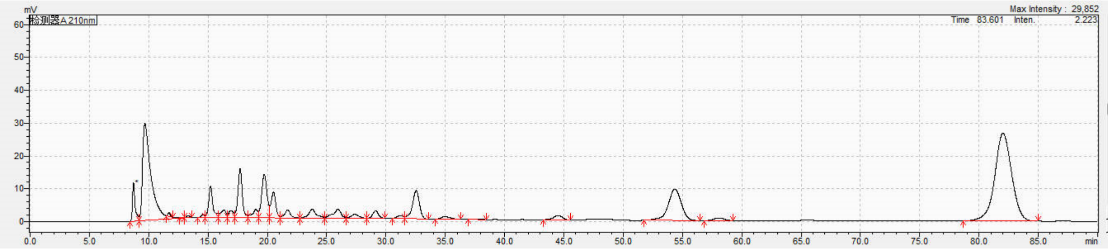

References:

[10] Wang, F.; Liu, B.; Cao, W.; Liu, L.; Zeng, F.; Qin, C.; Liang, C.; Huang, C.; Yao, S. Novel dual-action vanillic acid pretreatment for efficient hemicellulose separation with simultaneous inhibition of lignin condensation. *Bioresour. Technol.* **2023**, *385*, 129416. https://doi.org/10.1016/j.biortech.2023.129416.

[15] Liu, L.; Liu, B.; Li, X.; Wang, Z.; Mu, L.; Qin, C.; Liang, C.; Huang, C.; Yao, S. Mannitol assisted oxalic acid pretreatment of poplar for the deconstruction and separation of hemicellulose. *Ind. Crop. Prod.* **2023**, *200*, 116811. https://doi.org/10.1016/j.indcrop.2023.116811.

[16] Wang, S.; Liu, B.; Liang, J.; Wang, F.; Bao, Y.; Qin, C.; Liang, C.; Huang, C.; Yao, S. Rapid and mild fractionation of hemicellulose through recyclable mandelic acid pretreatment. *Bioresour. Technol.* **2023**, *382*, 129154. https://doi.org/10.1016/j.biortech.2023.129154.
[41] Tian, R.; Zhu, B.; Hu, Y.; Liu, Q.; Bian, J.; Li, M.; Ren, J.; Peng, F. Selectively fractionate hemicelluloses with high molecular weight from poplar thermomechanical pulp by tetramethylammonium hydroxide. International Journal of Biological Macromolecules 2024, 254, doi:10.1016/j.ijbiomac.2023.127499.
[42] Li, L.; Wan, Q.; Lu, Y.; Xia, L.; Xu, J.; Gou, J. Benzoic acid catalyzed production of xylose and xylooligosaccharides from poplar. Industrial Crops and Products 2024, 213, doi:10.1016/j.indcrop.2024.118460.
[12] Parsin, S.; Kaltschmitt, M. Processing of hemicellulose in wheat straw by steaming and ultrafiltration – A novel approach. Bioresource Technology 2024, 393, doi:10.1016/j.biortech.2023.130071.
[43] Xia, Q.; Zhang, L.; Zhan, P.; Tong, Z.; Qing, Y.; He, J.; Wu, Z.; Wang, H.; Shao, L.; Liu, N. Combination of microwave with acid deep eutectic solvent pretreatment for reed (Phragmites australis) fractionation. Renewable Energy 2024, 225, doi:10.1016/j.renene.2024.120286.
[44] Sun, Q.; Wang, B.; Huang, H.; Ma, C.-Y.; Ma, Y.; Shen, X.; Cao, X.; Sun, Z.; Zhang, L.; Yuan, T.-Q. Pressure-assisted hydrothermal pretreatment for biorefinery to enhance pulp production. Chemical Engineering Journal 2024, 487, doi:10.1016/j.cej.2024.150758.
